# News and Notes

**Published:** 1906-10

**Authors:** 


### NEWS AND NOTES.

---

Dr. Clem C. Green, of Atlanta, died September 3, aged forty-nine.

---

There is a rapid recession in the birth-rate of the cities of Germany.

---

Dr. Nicholas Senn, of Chicago, has returned from his trip to Africa.

---

Four civil suits have resulted from the poisoning with ice-cream at Salisbury Beach, Mass.

---

The health board of New York city in its recent investigation has found 229 insanitary bakeries.

---

Dr. A. W. Roaldes, of New Orleans, has been elected president of the American Laryngological Association.

---

It is estimated that there were 1,986 physicians present at the Toronto meeting of the British Medical Association.

---

Joaquin Albarran is to succeed J. C. F. Guyon, the famous French authority on affections of the genito-urinary organs.

---

Dr. H. T. Marshall, of Baltimore, has been appointed pathologist of the United States Bureau of Science at Manila.

---

Dr. John C. McVail, of Glasgow, Scotland, delivered the Lane medical lectures in San Francisco the latter part of August.

THE fourth Portuguese Congress for the Prevention of Tuberculosis will be held at Oporto, Portugal, from April 4th to 9th, 1907.

---

FOR seven weeks there has been no case nor death from yellow fever in Rio Janeiro, a condition not prevailing there for many years.

---

ACCORDING to the press dispatches, James M. Bringas, a mine-owner of Mexico, offers five millions of dollars for a cure for leprosy.

---

DR. FRANK M. RIDLEY, of LaGrange, Ga., has been appointed chief surgeon of the West Point route, to succeed Dr. Hunter P. Cooper.

---

IT is rumored that the plans are practically completed for the merging of the New Orleans Polyclinic and Tulane University of Louisiana.

---

DRS. DUNBAR ROY and Geo. H. Noble have returned from Toronto, Canada, where they read papers before the British Medical Association.

---

DR. J. H. LOVE killed Dr. D. M. Claitor August 28 in Natches, Miss. Dr. Love says that Claitor was of unsound mind, and had threatened his life.

---

DR. W. W. KEEN, of Philadelphia, was a recipient of one of the honorary degrees conferred at the four hundred and fiftieth anniversary of the University of Griefswald.

THE United States cruiser Columbia recently sailed for Boston from Porto Rico with 300 marines on board, of whom 165 were suffering from malarial infection contracted at Panama.

---

DR. ALEXANDER HUGH FERGUSON was recently informed by the Portuguese consul at Chicago that King Carlos, of Portugal, had awarded him a commandership in the Order of Christ of Portugal. This is the highest decoration the king of that country can bestow on any person outside of royalty.

---

THE Chicago Medical Society has appointed a committee of three pathologists to co-operate with the Chicago Commercial Association and the Illinois Manufacturers' Association in an investigation of the stockyards. One of the questions which it is especially desired to study is whether carcasses affected with local tuberculosis are fit for food after the diseased parts have been removed.

---

DR. W. S. ELKIN, dean of the Atlanta College of Physicians and Surgeons, will succeed the late Dr. Hunter P. Cooper in the chair of gynecology and obstetrics. Dr. Elkin has for many years been a constant associate, and connected in a business way with the late Dr. Cooper. Dr. W. S. Goldsmith has succeeded Dr. Cooper in the Elkin-Cooper Sanatorium, which is now to be called the Elkin-Goldsmith Sanatorium.

---

A memorial tablet was recently unveiled at Pavia, Italy, to the memory of the late surgeon, E. Bottini. Behind the tablet were immured a gold medal with the portrait of the deceased and an elaborate souvenir volume containing works by his pupils and friends, with an address on parchment—all of which had been prepared to present to the master on the occasion of the twenty-fifth anniversary of his assuming the chair of surgery at Pavia.

---

THE term "doctor" was invented in the twelfth century, about the time of the first establishment of universities. The first person upon whom this title was conferred was Irnerius, a professor of law at Bologna University. The title was created by Emperor Lohaire II, but was suggested by Irnerius himself. The term extended to the faculty of theology, and was first given by the University of Paris to Peter Lombard, the famous theologian. In 1329 the College of Asti conferred the first title of doctor of medicine upon William Gordenio.—*Medical Fortnightly*, September 10, 1906.

---

THE total number of medical students (matriculates) in the United States for the year ending June 30, 1906, was 25,204, a decrease of 943 below last year. Of this number, 23,116 were in attendance at the regular schools, 1,085 at the homeopathic, 644 at the eclectic, 110 at the physio-medical and 249 at the nondescript (unclassifiable) schools. The attendance at the regular schools shows a decrease of 1,003 below that of last year, a decrease of 546 below 1904 and a decrease of 1,814 below 1903. The number this year is the smallest since 1900. In the homeopathic school there was a decrease of 19 below the attendance of 1905, a decrease of 224 below 1904, there being a constant decrease since 1900, and the attendance for each of the past two years has been below that of 1880. The eclectic schools show a slight increase over last year, although the figures of each of the last two years are smaller than previous years since 1900. The physio-medical colleges had 110 this year, against 114 last year and 129 in 1904.—*Journal American Medical Association*, August 25, 1906.

